# Plant available phosphorus in soil as predictor for the leaching potential: Insights from long-term lysimeter studies

**DOI:** 10.1007/s13280-017-0975-x

**Published:** 2017-11-20

**Authors:** Holger Rupp, Ralph Meissner, Peter Leinweber

**Affiliations:** 1 Department Soil Physics, Helmholtz Centre for Environmental Research-UFZ, Lysimeter Station, Falkenberg 55, 39615 Altmaerkische Wische, Germany; 2 University of Rostock, Justus-von-Liebig-Weg 6, 18059 Rostock, Germany

**Keywords:** Agricultural management, Eutrophication, Fertilization, Free drainage, Lysimeter, Water protection

## Abstract

This study aimed to demonstrate the impact of phosphorus (P) mineral fertilization on topsoil P content and P leaching. We evaluated 83 datasets from 25 years from lysimeter experiments involving different cropping systems (winter crop, summer crop and autumn tillage, harvested grass) or unfertilized fallow, four types of soil texture, and three levels of applied mineral P fertilizer. A positive monotonic and significant correlation was indicated between P in the topsoil determined by the double lactate method (*P*_DL_) and the yearly flow-weight total (TP) concentrations in leachates with Spearman rank correlations *r*_s_ (*r*_s_ > 0.183) and probability (*p*) < 0.05. The present German recommended rates of P mineral fertilization are proposed insufficient to protect fresh and marine waters from undesired P pollution and eutrophication. A long-term reduction of excess soil P is urgent along with other measures to mitigate high P inputs to surface and ground waters.

## Introduction

Application of phosphorus (P) fertilizers should be adapted to soil P level both to enable optimal crop growth and to avoid undesired P leaching losses. In this context, relationships between the “optimal agronomic P level in soil” and resulting P leaching losses are insufficiently known, especially in the long term. Besides the broadly studied surface runoff (e.g. Sharpley 2016), leaching has been identified as an important P transfer pathway from soils to surface waters (Gachter et al. 1998). Total phosphorus (TP) leaching has been investigated in lysimeters that revealed a statistically significant relationship between soil P content and P leaching for sandy soils (Meissner et al. 1997). Phosphorus concentrations in lysimeter leachates and leaching losses were positively and significantly correlated with the contents of P extracted by NaHCO_3_ (*P*_Olsen_) and acid oxalate and the degrees of P saturation (Leinweber et al. 1999). For sandy loam soils (topsoil) under grass, the agronomic soil *P* tests “calcium acetate lactate extract” (*P*_CAL_), “double lactate extract” (*P*_DL_), Mehlich-3 *P* test and *P*_Olsen_ enabled reasonable predictions of P in lysimeter leachates in the same study. Under arable use, factors such as fertilization, management intensity, depth of tillage and irrigation resulted in non-significant correlations between soil P concentrations and P in leachate (Godlinski et al. 2004). These observations strongly call for deriving site- and management-specific coefficients to estimate potential P losses from the soil P status.

In UK field studies, the soil P concentrations in tile drain waters were low at < 60 mg *P*_Olsen_ kg^−1^ in the topsoil. Above this soil P content (termed the “change-point”), P in soluble forms in drainage waters increased rapidly along with the soil P content (Heckrath et al. 1995; Brookes et al. 1997; Hesketh and Brookes 1998). As a result of column and lab experiments, Maguire and Sims (2002a, b) found also a change point, below which P leachate increased slowly per unit increase in soil test P, and above which leachate dissolved reactive phosphorus (DRP) increased rapidly. More recently, Wuenscher et al. (2016) reported a similar correlation between the amounts of labile P fractions and P leaching losses for soils representing different soil textures, land uses and management practices. In that review, a range of different extraction in the laboratory and lysimeter studies with P concentration in drainage agreed to the “change points” that were estimated by 0.01 M calcium chloride (CaCl_2_) extraction. In partial disagreement to the above-cited works, Djodjic et al. (2004) found no general correlation between *P*_Olsen_, ammonium lactate extracted-P (*P*_AL_) and P concentrations leached from 1-m-deep columns with five different Swedish agricultural soils. Studies using similar lysimeters revealed that it may take a long time to reduce soil P concentrations and P leaching even after P application has ceased (Svanbäck et al. 2015). Upscaling of results from field trials to the catchment scale introduces much insecurity in predictions because P loss from agricultural land is controlled by factors which are independent on added annual P surpluses and soil P contents (Edwards and Withers 1998). Large P loads to British catchments were mainly related to factors such as soil clay content, general level of precipitation, P fertilization and manure application (Edwards and Withers 1998). Also the kind of manure, especially the proportion of water soluble P and the proportions of annual crops in the catchments, can be important factors (Moog and Whiting 2002; Kyllmar et al. 2006; Stutter et al. 2008). There, for establishing region-specific best management practices (BMPs), the relationships between soil properties/management, soil P status and the P loss-risk must be well established from long-term datasets. In flat areas with shallow groundwater levels, direct observation of P leaching using lysimeters is valuable, while simple index methods are seriously limited in estimating the P leaching (Schoumans et al. 2013).

Target values for TP concentrations in water resources in German and European environmental legislation distinguish between running waters (e.g. river, brooks, ditches) and stagnant waters (lakes). No TP limits are legally established for groundwater, but the target value of 0.5 mg L^−1^ is currently in discussion. In Germany, new orientation values for a good ecological status were recently formulated (OGewV 2016). These values were used in context with European water quality standards (Phillips and Pitt 2015). Efforts in Germany are focused on preventing eutrophication by achieving at least the “good ecological status” for surface waters according to the regulations of the Water Framework Directive (European Union 2000). Accordingly, the TP concentrations in streams should not exceed a concentration of 0.1 mg L^−1^. German national classification and recommendations for soil *P* test values are based on P extracted by calcium-acetate lactate (*P*_CAL_) (VDLUFA 2015) and recommendations related to surface water on OGewV (2016).

The objectives of the present study were (i) to evaluate the impact of P fertilization related to the soil P contents on P leaching in several agricultural systems and (ii) to discuss the long-term potential impact on surface and ground water resources with the background on currently recommended P fertilization and soil P status in Germany.

## Materials and methods

This study is based on 83 non-weighing gravity-flow (free drainage) lysimeters (NWLYS) which are located at the Helmholtz Centre for Environmental Research-UFZ lysimeter station at Falkenberg, northern part of Germany (52°51′N, 11°48′E). Lysimeters with representative land usage and soil texture for the Elbe river catchment were selected for this study.

Climatically, the lysimeter site is assigned to the temperate zone of central Europe within the transition zone from maritime to continental climate. The lysimeter station is equipped with a meteorological station. The daily amount of precipitation was measured with a standard Hellmann-rain gauge (1 m above ground level). Precipitation averages 570.8 mm per year (1991–2015; Falkenberg), with maximum precipitation occurring during June and July. Mean annual temperatures range from 7.3 to 10.1 °C (1991–2015) with occasional freezing in winter months.

The simple NWLYS type is used often in Germany and other central European countries for applied research on land management and its impact on drainage water quantity and quality (Lanthaler and Fank 2005; Weihermueller et al. 2007). They were constructed in the form of a sheet steel vessel with a quadratic surface area of 1 m^2^ and a total depth of 1.25 m. After the installation at the lysimeter station, a 25-cm-thick filter layer (sand over gravel over stone gravel) was placed at the bottom of the vessels. A PVC-drainage pipe (inner diameter 63 mm) was installed inside the filter layer to collect the seepage and to discharge it into a storage tank located at the lysimeter cellar (Meissner et al. 2010). In the year 1981, all lysimeters were filled manually with disturbed soil material from four different agricultural sites in eastern Germany, representing sand (S), loamy sand (LS), loam (L) or silty loam (Si) both in topsoils and subsoils (Table 1) of the Elbe river catchment (Godlinski et al. 2004). In order to resemble the original soil structure of the sites, the soil was excavated in two layers (topsoil 0–30 cm and subsoil 31–100 cm), stored separately and then transported to the Helmholtz lysimeter station and filled manually in layers in the lysimeter vessels. The layers were compacted manually to obtain a bulk density as in the field site. After this filling procedure, the lysimeters were irrigated to accelerate the setting process (approximately 100 mm irrigation water per year; Meissner et al. 2010). Saturated hydraulic conductivity (*K*_S_) measured in 1981 (before filling into the lysimeters) varied only slightly more than a 10-potency among the different soil texture types. For all soils, the content of total organic carbon as well as soil pH was low (Table 1). The initial lysimeter trial was started in 1983. Results presented here are from the period 1991–2015.

**Table 1:** Basic parameters of the lysimeter soils. Soil texture class (WRB 2006), soil texture with sand (2.0–0.06 mm), silt (0.06–0.002 mm) and clay (< 0.002 mm). Bulk density (ζ_d_), saturated conductivity (*K*_s_), soil pH, total organic carbon (TOC) in topsoil (0–30 cm) and subsoil (31–100 cm) in the lysimeters measured at the agricultural sites from which the lysimeter soils were taken

Soil texture	Sand (S)		Sandy loam (SL)		Loam (L)		Silty loam (Si)	
Layer	Topsoil	Subsoil	Topsoil	Subsoil	Topsoil	Subsoil	Topsoil	Subsoil
Sand (%)	88.2	91.2	73.6	75.2	50.4	57.6	4.7	2.8
Silt (%)	6.7	6.7	14.3	17.4	37.5	20.3	74.8	80.9
Clay (%)	5.1	2.1	12.1	7.4	12.1	22.1	20.5	16.4
ζ_d_ (g cm^−3^)	1.3	1.7	1.5	1.8	1.6	1.8	1.2	1.2
*K*_s_(cm d^−1^)^a^	106	200	21	43	21	12	32	45
pH_KCl_^b^	5.6	6.0	4.8	5.6	6.3	6.5	6.9	7.0
TOC (%)	0.7	0.0	1.1	0.2	1.0	0.1	1.7	1.8

^a^Stationary procedure

^b^Potassium chloride method

### Cultivation of the lysimeter soils

Table 2 gives an overview of different management regimes representing grassland or plough land together with mineral P fertilizer applications. Manure in liquid or solid form was applied in two of the experiments. The cultivation was typical for agricultural production in the reunified Germany in 1991 while the fertilization covered a wider range than in practical farming for experimental reasons. Crop rotations contained clover and catch crops to fit the requirements of European policy, subsidies from common agricultural reform and governmental aid. The established crop rotations have been maintained since this time. The dependency of soil *P*_DL_ content on the mineral P fertilization level was studied using the lysimeter trials Organic Farming (OF), Best Management practice (BMP), Different Mineral Fertilization (DMF), Fallow and Different Grassland Management (DGM). The lysimeter trial DMF comprises 24 lysimeter vessels representing all four types of soil texture. Three lysimeter vessels of each soil texture class were sown and tilled each year and three were permanent grassland (Table 2). In our lysimeter study, we used catch crops in OF, such as a mixture of corn and sun flowers which were harvested in late autumn and removed from the lysimeters.

**Table 2:** Overview on the experimental lysimeter management practices and P fertilization. *W.* winter

Land management	Designation of experiment	Number of lysimeters	Soil texture	Crop rotation	Mineral P fertilization (kg ha^−1^)	Organic P fertilization (kg ha^−1^)
Arable land	Organic farming (OF)	7	LS	W. Wheat & catch crop	0	14^a^
				Pea & catch crop.	0	–
				W. Wheat & catch crop	0	14^a^
				Oats & underseed	0	–
				Clover mixture	0	–
				Potatoes	0	–
Arable land	Best management practice (BMP)	38	LS	W. Wheat & catch crop	20	–
				Potatoes	20	90^b^
				W. Barley & catch crops	20	–
				Corn	20	–
				Sugars beets	20	75^b^
Arable land	Different mineral fertilization (DMF) level 50%	4	LS, S, L, Si	W. Wheat	12.5	–
				W. Barley & catch crops	12.5	–
				Oats & underseed	12.5	–
				Clover mixture	15	–
Arable land	Different mineral fertilization (DMF) level 100%	4	LS, S, L, Si	W. Wheat	25	–
				W. Barley & catch crops	25	–
				Oats & underseed	25	–
				Clover mixture	30	–
Arable land	Different mineral fertilization (DMF) level 150%	4	LS, S, L, Si	W. Wheat	37.5	–
				W. Barley & catch crops	37.5	–
				Oats & underseed	37.5	–
				Clover mixture	45	–
Grassland	Fallow	6	LS	Annually 1 cultivation cut	0	–
Grassland	Different grassland management (DGM)	6	LS	Extensive Grassl., 2 cuts	10	–
				Standard Grassl., 3 cuts	25	–
				Intensive Grassl., 4 cuts	40	–
Grassland	Different mineral fertilization (DMF) level 50%	4	LS, S, L, Si	Grass, 4 cuts	20	–
Grassland	Different mineral fertilization (DMF) level 100%	4	LS, S, L, Si	Grass, 4 cuts	40	–
Grassland	Different mineral fertilization (DMF) level 150%	4	LS, S, L, Si	Grass, 4 cuts	60	–

^a^Application rate according to the nitrogen content—2 applications of each 40 kg N ha^−1^, highly variable P content 10–700 mg L^−1^

^b^Farmyard manure, P content 1–3.1 kg P t^−1^

The mineral fertilizer “Triple Super Phosphate” (Helm AG, Hamburg, Germany) containing 20% total P was applied for mineral P fertilization of the lysimeters. The granulated fertilizer was annually spread (single treatment) in early spring at the beginning of the growing season according to the experimental schedule (without consideration of soil P contents). Crop protection products (both herbicides and pesticides) were not used on the lysimeters. The crop residues were tilled down into the soil after harvest and weeds were treated mechanically by tilling with cultivator.

The lysimeters were irrigated from 1991 until 2003 according to the plant physiological requirements for yield maximization. Depending on crop and specific climate conditions, up to 350 mm water were additionally applied. This irrigation regime was changed in 2004. From this date onwards, the crops were irrigated exclusively for the purpose of safeguarding plant stocks, which resulted in a significant reduction in the amount of irrigation water applied (up to 50 mm annually).

### Soil sampling

The topsoil of the lysimeters was sampled 10–18 times within the 25-year-long experimental period. Sampling was regularly carried out at the beginning of vegetation period (end of February until mid of March) by randomly taking several small samples and mixing to a representative composite sample. Soil *P*-test values were determined using the *P*_DL_ test (VDLUFA 1991) that is mostly used to classify the soil P status of agricultural land in the Elbe river catchment and other parts of eastern Germany and to release P fertilizer recommendations to farms. In other parts of Germany, extraction with the *P*_CAL_ test is used for *P*-test value classification (VDLUFA 2015). From intercalibration between the two methods, the *P*_DL_ values were converted to *P*_CAL_ (van Laak and Buczko 2016).

$$ P_{\text{CAL}} = \, 1.78 \, + \, 0.63 \, \times \, P_{\text{DL}} \left( {R^{2} = \, 0.70} \right). $$

VDLUFA classifies the soil P status according to Table 3 into five classes from very low (*A*) until very high (*E*) among which the class *C* is the target level of plant available soil P. In fact, a recent update of this scheme means a reduction of the target soil *P*-test values by approximately factor 1.7: class *C* = 45–90 mg *P*_DL_ kg^−1^ (VDLUFA 2015).

**Table 3:** Comparison of former and presently recommended *P*_CAL_ and *P*_DL_ contents in soils according to the recommendations of the VDLUFA and TP orientation values for water quality according to the German OGewV (2016) and European quality standards (Phillips and Pitt 2015)

Classes of soil *P*-test values	Former VDLUFA recommendation (Kerschberger et al. 1997) (mg *P*_CAL_ kg^−1^ soil)	Former VDLUFA recommendation (mg *P*_DL_ kg^−1^ soil)^a^	Novel VDLUFA recommendation (VDLUFA 2015) (mg *P*_CAL_ kg^−1^ soil)	Novel VDLUFA recommendation (mg *P*_DL_ kg^−1^ soil)^a^
A (very low)	20	30	15	20
B (low)	45	70	30	40
C (optimum, recommended)	90	140	60	90
D (high)	150	240	120	190
E (very high)	> 150	> 240	> 120	> 190

Surface waters	Orientation values “very good ecological status” (OGewV 2016) TP (mg L^−1^)	Orientation values “good ecological status” (OGewV 2016) TP (mg L^−1^)	Span of European quality standards (Phillips and Pitt 2015) TP (mg L^−1^)	Median of European quality standards (Phillips and Pitt 2015) TP (mg L^−1^)
Rivers	≤ 0.05	≤ 0.10	0.01–0.5	0.10
Lakes	≤ 0.017–0.035	≤ 0.025–0.045	0.005–0.1	0.03

^a^Converted according to (van Laak and Buczko 2016)

### Leachate sampling, water analyses and assessments

Lysimeter leachates were continuously sampled in the storage tanks when discharge occurred (predominantly in the months of November–April). Samples were taken once a month and analyzed in the UFZ laboratory for concentrations of TP according to DIN 38405-9 (1983) (photometrically by the molybdate-blue method). We calculated monthly loads based on monthly TP concentration and amount of seepage water, which were finally used for the calculation of average annual TP concentrations and annual loads. Average annual TP concentrations for a period of 25 years (1991–2015) were used to evaluate the different land management systems and fertilization levels. TP concentrations were compared with standard target values for water resources in German and European environmental legislation to assess our lysimeter leachates. In Germany, new orientation values for good ecological status in surface waters were recently formulated (OGewV 2016). These values were used in combination with European water quality standards (Phillips and Pitt 2015). Efforts in Germany are focused on preventing eutrophication by achieving at least the “good ecological status” for surface waters according to the regulations of the Water Framework Directive (European Union 2000). Threshold TP concentrations for the quality classes of both, rivers and lakes, are compiled in Table 3 because leached P can reach both types of water bodies.

### Statistics

Descriptive statistical methods with linear regression function were applied for data assessment and performed using the software package ORIGIN (OriginLab Corporation, Northampton, USA). Since measured values were not normally distributed, Spearman’s rank values (*r*_s_) were estimated. This coefficient is robust against outliers. Furthermore, the Kruskal–Wallis test (One-way ANOVA on ranks) as a non-parametric method was used for testing whether lysimeter data originate from the same distribution.

## Results and discussion

### Mineral P fertilization effect on soil *P*_DL_ content

**Fig. 1:**
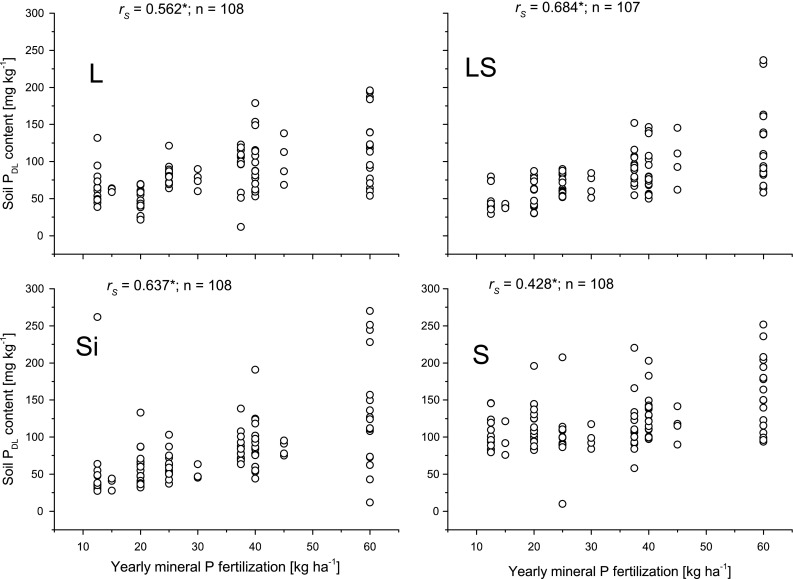
Plot of soil *P*_DL_ contents versus yearly mineral P fertilization for soil texture classes loam (L), loamy sand (LS), silt (Si) and sand (S)

Soil *P*_DL_ related to yearly mean P fertilization is presented for each texture since soil *P*_DL_ contents showed significant differences between the four texture types *p* < 0.001 (Kruskal–Wallis–ANOVA) (Fig. 1). Regular application of P mineral fertilizers had a significant positive influence on the *P*_DL_ contents but there were differences between soil texture classes. The dependency of *P*_DL_ on mineral fertilization was apparent for the LS lysimeters (*r*_s_ = 0.684; *n* = 107). For the S lysimeters this relationship was weaker (*r*_s_ = 0.428; *n* = 108), and texture classes L and Si lysimeters ranged in between. Some soils showed comparatively high levels of *P*_DL_ contents, as expected from decades of fertilization with up to 60 and up to 90 kg P ha^−1^ a^−1^ of manure P (Table 2).

**Fig. 2:**
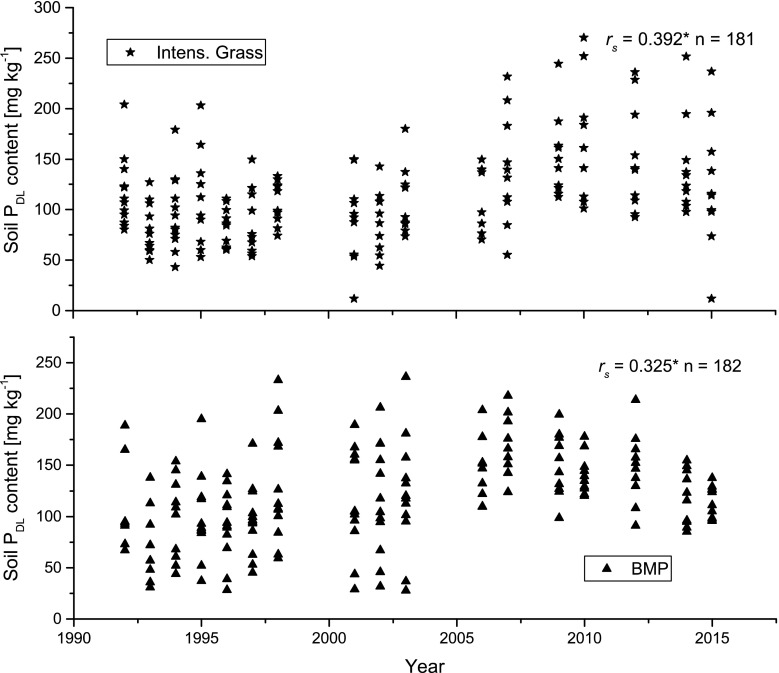
Temporal change of soil *P*_DL_ contents within the lysimeter trials Best Management Practice (BMP) and Intensive grassland (Intens. Grass) for the 25 years observation period 1991–2015

The soil *P*_DL_ increased significantly on lysimeters managed conventionally according to BMP during the long-term investigation period of 25 years (*r*_s_ = 0.325 and p < 0.001). Intensive grassland management also resulted in a statistically significant increase in soil *P*_DL_ contents (*r*_s_ = 0.392 and p < 0.001) (Fig. 2). In these two management systems, mineral fertilization of up to 60 kg P ha^−1^ and organic fertilization of up to 90 kg P ha^−1^ resulted in accumulation of P in the topsoil layer within the 25 years of experimental period. Similarly, Schoumans et al. (2014) reported a tendency to P accumulation in the topsoil layer, involving an increased risk of P leaching losses. Therefore, an annual P fertilization without considering the actual soil P status has to be avoided. Furthermore, freezing catch crops (oilseed radish) as implemented in the BMP crop rotation, can become a source of P losses to water resources after exposure to freezing–thawing cycles (Liu et al. 2014).

**Fig. 3:**
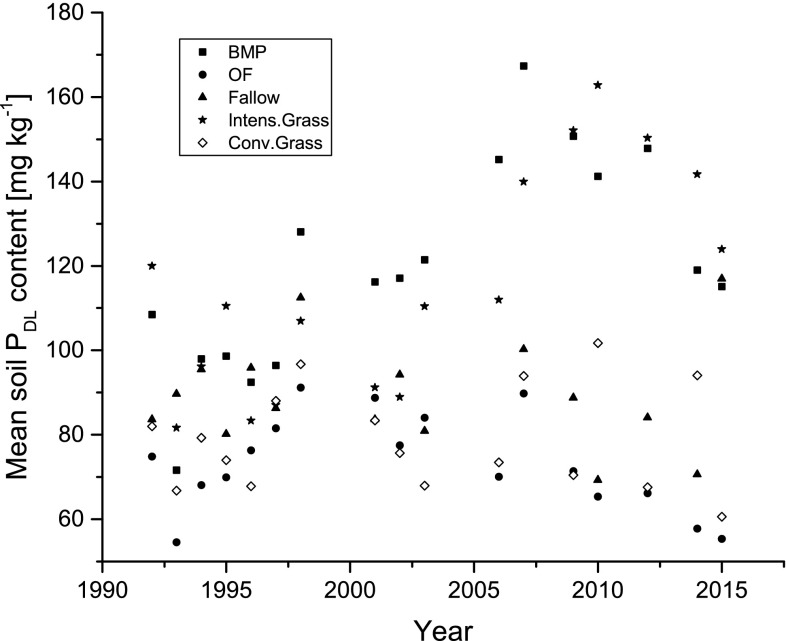
Temporal course of mean soil *P*_DL_ content (1991–2015) for selected variants of land use in a lysimeter trial. *BMP* best management practice, *OF* organic farming, Fallow, *Conv. Grass* conventional grassland, *Intens. Grass* intensive grassland

Lysimeters with sandy soils in trial DMF showed in the land management system grassland the weakest but still significant dependency of *P*_DL_ on the annual mineral P fertilization. For arable land this statistical relationship does not exist (Table 4). The highest *r*_s_ values (0.74–0.78) between mineral P fertilization and soil *P*_DL_ content were estimated for LS and Si lysimeters with arable land, yearly tilled and amended with varying amounts of mineral fertilizers (DMF). Conventional farming with moderate organic (up to 90 and up to 75 kg P ha^−1^ farmyard manure for potatoes and sugar beets, respectively, cf. Table 2) and mineral fertilization according to the BMP scheme resulted in a mean soil *P*_DL_ content of 110 mg kg^−1^ (standard deviation: *s*_d_ = 45 mg kg^−1^; *n* = 473). Long-term fallow after previous intensive management also resulted in a reduction of soil *P*_DL_ contents compared to BMP (Fig. 3). Here the *P*_DL_ contents were reduced in comparison to BMP at 95.6 mg kg^−1^ (*s*_d_ = 31.9 mg kg^−1^; *n* = 77). These *P*_DL_ contents were still comparatively high. The unploughed fallow vegetation cover was cut once per year according to the experimental scheme. The plants may have taken up P from deeper soil layers and remained on the soil surface. Therefore, an effective reduction of the soil *P*_DL_ contents was not achieved with fallow. Conventionally managed grassland with 2–3 annual cuts (DGM, extensive and conventional grassland), unploughed and receiving 10–20 kg P ha^−1^ year^−1^, had 77 mg *P*_DL_ kg^−1^ (*s*_d_ = 20.2; *n* = 72). Organic farming represented by 7 lysimeters had a low mean *P*_DL_ content of 62 mg kg^−1^ (*s*_d_ = 16.5; *n* = 117). The required target soil *P*_DL_ content in class *C* of 90 mg kg^−1^ (VDLUFA 2015) was achieved only in organically managed lysimeters without mineral P fertilization and in extensively and moderately conventionally managed grasslands, whereas the other managements resulted in soils with unacceptably high P status.

**Table 4:** Linear regression functions *y* = *a* + *b* × *x* describing the dependency of *P*_DL_ (year, mg kg^−1^) from the annual P fertilizer amounts ranging between 12.5 and 60 kg ha^−1^ for several lysimeter trials and differently textured soils; *R*_s_ = correlation coefficient (Spearman); *p* = probability value; *n* = number of data pairs

Designation of experiment	Land management	Texture	X	Y	*a*	*b*	*R*_S_	*p* value	*n*
Best management practice (BMP)	Arable land	LS	PDL	P fertiliz.	8.94	0.07	0.2721*	< 0.001	469
Different grassland management (DGM)	Grassland	LS	PDL	P fertiliz.	3.93	0.24	0.5757*	< 0.001	106
Different mineral fertilization (DMF)	Grassland	L	PDL	P fertiliz.	19.70	0.233	0.7180*	< 0.001	54
Different mineral fertilization (DMF)	Arable land	L	PDL	P fertiliz.	10.33	0.20	0.4986*	< 0.001	54
Different mineral fertilization (DMF)	Grassland	LS	PDL	P fertiliz.	21.32	0.21	0.6278*	< 0.001	53
Different mineral fertilization (DMF)	Arable land	LS	PDL	P fertiliz.	6.18	0.29	0.7433*	< 0.001	54
Different mineral fertilization (DMF)	Grassland	Si	PDL	P fertiliz.	25.86	0.15	0.5362*	< 0.001	54
Different mineral fertilization (DMF)	Arable land	Si	PDL	P fertiliz.	5.58	0.34	0.7752*	< 0.001	53
Different mineral Fertilization (DMF)	Grassland	S	PDL	P fertiliz.	15.55	0.18	0.4235*	< 0.01	54
Different mineral fertilization (DMF)	Arable land	S	PDL	P fertiliz.	20.04	0.06	0.2122	> 0.05	54
Different mineral fertilization (DMF)	Grassland	S, LS, Si, L	PDL	P fertiliz.	23.50	0.163	0.5073*	< 0.001	216
Different mineral fertilization (DMF)	Arable land	S, LS, Si, L	PDL	P fertiliz.	17.06	0.113	0.4329*	< 0.001	216

*Significant relations on the probability level ≤ 0.05

### Total P concentrations in lysimeter leachates as functions of soil *P*_DL_ contents

**Fig. 4:**
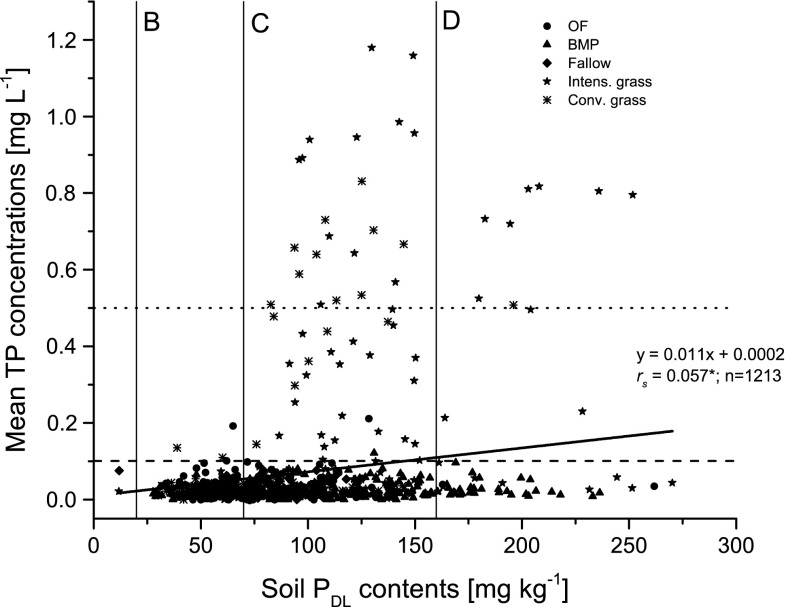
Plot of mean annual TP concentrations in leachates for selected forms of land usage versus soil *P*_DL_ contents. *Dotted horizontal line* indicates the upcoming German groundwater P threshold of 0.5 mgP L^−1^. The dashed line indicates concentrations > 0.1 mgP L^−1^ which corresponds to a critical exceedance of orientation values for rivers (OGewV 2016). *Vertical lines* indicate the limits of novel P contents according to VDLUFA (2015) fertilizer recommendations. *BMP* best management practice, Fallow, *Conv. Grass* conventional grassland, *OF* organic farming, *Intens. Grass* intensive grassland

The plot of all values of soil *P*_DL_ contents versus the corresponding average TP concentrations (Fig. 4) resulted in relatively high *P*_DL_ contents that often exceeded the range for the non-recommended class E (very high) of 190 mg kg^−1^ soil (VDLUFA 2015). This indicates an undesired P accumulation, and that corresponding mean annual TP concentrations in leachates also tend to be high. The long-term average TP concentration amounts to 0.066 mg L^−1^ (median 0.024 mg L^−1^) but maximum values of up to 1.179 mg L^−1^ point to a distinct pollution risk for running waters since the TP concentrations are far above the targeted values given by OGewV (2016) and Phillips and Pitt (2015). Additionally, these maximum values of TP concentration also exceeded the aforementioned upcoming orientation value for groundwater quality of 0.5 mg P L^−1^ bearing a risk for groundwater pollution. Intensively and conventionally used grassland showed the highest mean TP concentrations, often exceeding the orientation value for good ecological status of rivers (OGewV 2016). Although appearing weak, the relationship between mean annual TP concentrations in leachates and soil *P*_DL_ contents was statistically significant (*r*_s_ = 0.1072, *p* < 0.001, *n* = 1174). A similarly weak significant correlation between mean annual TP concentrations in leachates and the *P*_DL_ contents in soil was estimated for grassland (*r*_s_ = 0.348, *p* < 0.001, *n* = 393). The general lack of strong statistical relationships for ploughed arable land with yearly different crops can be explained by the variety of experimental scenarios (crop rotations and tillage measures).

Lehmann et al. (2005) studied the relations between soil P content and leachates from small lysimeters after long-term manure application. They discovered that the ability of these soils to retain additional P was low and equilibrium leachate concentrations of total dissolved P (TDP) were high. In lysimeter studies, Djodjic et al. (2004) found no general relation between P concentrations and soil test P of the topsoil for soil of different texture classes. They concluded that water transport mechanisms through the soil and subsoil properties seemed to be more important for P leaching than soil test P value in the topsoil. On the other hand, Ulén et al. (2016) clearly demonstrated increasing concentrations of DRP in tile drains along increasing soil *P*-test values in the topsoil. Weak correlations between DRP in lysimeter leachates and the agronomic soil tests Mehlich-3 P and *P*_Olsen_ were reported for organically managed soils from Ontario (Zheng et al. 2015). Our above relationships between the two factors mineral P fertilization and soil P related to TP appeared too weak to derive reliable predictions of leachate TP concentrations based on soil P values and texture since the *r*_s_ value was low (*r*_s_ = 0.010). The significance was high (*p* < 0.001) due to the large number of datasets/pairs included.

To achieve the orientation values for good ecological status in lakes, the TP concentrations should not exceed 0.045 mg L^−1^. The new reduced target range for soil *P*_DL_ content in class C “optimum recommended” an upper limit of 90 mg kg^−1^ (*P*_DL_) (VDLUFA 2015), and this often corresponds with mean annual TP concentrations > 0.045 mg L^−1^ according to our study. For instance, the function for soil *P*_DL_ contents implicated a TP concentration of about 0.07 mg L^−1^ corresponding with the class C upper limit of 90 mg kg^−1^. This means that Germany defined an agronomic optimum value of 90 mg kg^−1^ which essentially bears a risk of increasing freshwater P pollution.

The long-term average P concentrations of the management forms OF, BMP, Fallow and conventionally used grassland were at an equal level. Organic Farming without additional mineral P fertilizer had a comparatively low average TP concentration of 0.026 mg L^−1^. But Hansen et al. (2001) pointed out that organic farming carries a high risk of P leaching in fields receiving or producing sources of organic matter (animal manure, green manure, catch crops, clover-grass, etc.) that raise the mobility of P in the soil. Highly variable P concentrations in liquid manure, ranging from 10 to 700 mg P L^−1^, and the application according to the nitrogen content of the liquid manure possess an additional risk for P accumulation and leaching. Conventional arable land management with mineral P fertilization of 20–45.5 kg ha^−1^ corresponded with TP concentrations of 0.027 mg L^−1^. This was in the same range with the average TP concentrations in leachates from long-term fallow of former intensively managed arable land (0.030 mg L^−1^) and extensively and moderately conventionally managed grasslands (0.029 mg L^−1^). Lysimeters with intensive grassland management receiving 40–60 kg ha^−1^ a^−1^ mineral P fertilization showed a significant increase in the long-term average TP concentration to 0.154 mg L^−1^. This very high concentration is most probably a result of a fertilization solely to meet the need of nitrogen for the crops essentially resulting in a surplus of P. However, grass plants have a high need for P which in organic farming is met by animal and green manure addition. Thus, especially the management variants OF, BMP, long-term fallow and conventionally managed grassland meet the orientation value for “good ecological status” of lakes (Phillips and Pitt 2015; OGewV 2016).

### Lysimeter seepage

**Fig. 5:**
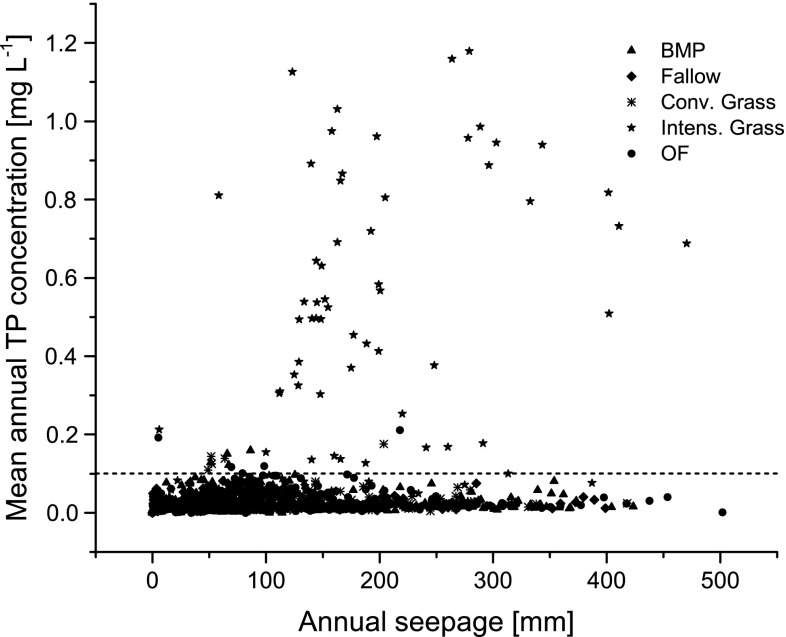
Mean annual TP concentrations in leachates versus annual seepage. The dashed horizontal line indicates concentrations of >0.1 mgP L^−1^ which corresponds to a critical exceedance of orientation values for rivers (OGewV 2016). *BMP* best management practice, Fallow, *Conv. Grass* conventional grassland, *OF* organic farming, *Intens. Grass* intensive grassland

In addition to soil *P*_DL_ contents, the annual seepage affects the TP concentration. This effect became especially evident for intensive grassland usage, receiving high P mineral fertilization and intensive irrigation until 2003 that has been oriented on yield maximization (Fig. 5). Seepage and annual mean TP concentrations were strongly correlated for intensively used grassland (*r*_s_ = 0.453, *p* < 0.001). For BMP and OF, this relationship was weaker with correlation coefficients of *r*_s_ = 0.104 (*p* < 0.05) and *r*_s_ = 0.169 (*p* < 0.05), respectively.

Based on 2058 data pairs (total available dataset of all lysimeters), the average TP concentrations and measured annual seepages were statistically significantly related (*r*_s_ = 0.20, *p* < 0.001). Furthermore, if we consider data points above a critical TP concentration >0.1 mg L^−1^ that corresponds to a critical exceedance of orientation values for rivers (OGewV 2016), a strong linear correlation with the seepage (*r*_s_ = 0.358; *n* = 68) becomes evident (Fig. 5). Djodjic et al. (2004) considered water transport mechanism through the soil and subsoil as highly important for P leaching. Intensive precipitation events may mobilize excess P by internal erosion, most likely along preferential flow pathways. In this line of evidence, Zimmer et al. (2016) reported that critical hydrological events at the field scale caused the transfer of half of the mean annual P load into the Baltic Sea from north-eastern German catchments during a few days.

## Conclusions

This study clearly demonstrated the need for long-term studies evaluating the relation between plant available P in soil and P concentration in leachates to predict the P leaching potential from agriculturally managed soils. Agricultural management according to BMP, Fallow and DGM practices for 25 years had a tendency to impact soil *P*_DL_ contents. The conventional agricultural management with static P fertilization and the intensive grassland management (without consideration of actual soil *P*_DL_ contents) resulted in a significant P accumulation in topsoil. Therefore, farmers should be supported to change their fertilization strategy and to reduce external inputs of P fertilizers to get the topsoil P in balance.

The mean annual TP concentrations are relatively imprecise in disclosing the relationships between hydrology and P losses. Therefore, in the forthcoming studies we will evaluate the monthly data for the 25-year-experimental period to detect event-based elevated TP concentrations. These will lead to a better understanding of the P leaching and lay a basis for developing technical measures to capture inevitable P loads at field edge or drainage outlets. Such measures appear necessary to achieve the goals of international commitments on conserving freshwater and marine ecosystems.

Since even the actually reduced P levels of VDLUFA (2015) are insufficient to completely prevent undesired P leaching losses and transfers to waterways, the present-day P fertilizer recommendations should be questioned and critically evaluated. Because international agreements such as the Baltic Sea Action Plan (HELCOM 2014) bind Germany by contract to drastically reduce the P inputs to the Baltic Sea, diffuse losses from agricultural fields, among which P leaching is a key process in flat to undulated Pleistocene landscapes of northern Germany, must be minimized. Therefore, lower soil P levels throughout the agro-ecosystems have priority over crop yield and production maximization.
